# Cattle are more motivated for a high-concentrate diet than Sudan grass hay, despite low reticulorumen pH

**DOI:** 10.1093/jas/skae049

**Published:** 2024-02-24

**Authors:** Rachael E Coon, Cassandra B Tucker

**Affiliations:** Center for Animal Welfare, Department of Animal Science, University of California, Davis, Davis, CA 95618, USA; Center for Animal Welfare, Department of Animal Science, University of California, Davis, Davis, CA 95618, USA

**Keywords:** beef cattle, electric current, forage, motivation, pH

## Abstract

Subacute ruminal acidosis (**SARA**) is characterized by chronic low ruminal pH, and occurs for feedlot cattle fed high-concentrate diets. Forages slow digestion and reduce acid production. We aimed to assess how motivated finishing cattle are to access forage (Sudan grass hay, **SG**) via their willingness to interact with an electrified barrier. Reticulorumen pH was measured to relate the results to digestive health. Twenty-eight animals fed a high-concentrate ration ad libitum had access to 4 L of one of two treatments (*n* = 14/treatment) fed 1×/d behind a barrier: 1) SG or 2) an additional offering of the normal ration (total mixed ration [**TMR**]). To access their treatment, the steer voluntarily pushed his muzzle against an electrified barrier. The electrical current was increased exponentially every 24 h (0, 156, 312, 625, 1,250, 2,500, 5,000 µA) until the animal ceased accessing it. Visits to the treatment were recorded continuously 24 h/d and reticulorumen pH was measured every 10 min. Time with a reticulorumen pH below 5.8 was 348 ± 101 and 280 ± 76 min/24 h for SG and TMR animals, respectively; these durations meet the criterion for SARA. However, animals with access to SG were less likely to advance to the next current than TMR animals (*P* < 0.01) and were approximately 3× less willing to interact with higher currents than TMR (mean maximum current touched: 469 ± 169 and 1,380 ± 254 μA, respectively, mean ± SE, *P* = 0.01). Lower motivation to access SG was further demonstrated through fewer visits to the SG (2.4 ± 0.4 vs. 5.3 ± 0.6 #/d, *P* < 0.01), and less SG consumed than TMR (32.0 ± 0.1 vs. 74.0 ± 0.0 %/d, *P* < 0.01, measured as % due to weight differences of SG and TMR). Overall, finishing cattle valued the TMR more than SG, likely because of differences in the quantity offered, palatability, and familiarity. When rumen health was considered, SG animals visited more often (*r* = 0.5, *P* = 0.09) and showed fewer failed attempts (*r* = −0.5, *P* = 0.06) to access forage as the severity and duration of pH depression below 5.6, for example, increased. No measures of treatment use were related to pH depression for TMR animals (*P* ≥ 0.31). These findings provide evidence that cattle are motivated for Sudan grass hay when experiencing chronic low reticulorumen pH. However, they also contribute to the mixed evidence about the motivation for forage in this life stage, because, overall TMR was valued more highly than SG. Despite widespread pH depression, TMR cattle contrafreeloaded for additional concentration, demonstrating unexpectedly high motivation for this resource.

## Teaser

Finishing cattle willingly touch an aversive electric current to access additional grain despite experiencing chronic low reticulorumen pH.

## Introduction

The diet fed to 48% of American feedlot cattle is composed of ≥76% concentrate or grain on a dry matter (**DM**) basis (USDA, 2013). For an animal that has evolved to eat a forage-based diet, this high intake of grain can have detrimental consequences for rumen function (Krause and Oetzel, 2006). Acute ruminal acidosis, which can occur because of excessive acid production in the rumen caused by high intake of grain, can result in systemic illness (e.g., metabolic acidosis (Hernández et al., 2014)) and death (Nagaraja and Lechtenberg, 2007). The less severe form of the disorder is subacute ruminal acidosis (**SARA**), which can lead to inconsistent dry matter intake (**DMI**), reduced weight gain, rumenitis (Owens et al., 1998), liver abscesses (Nagaraja and Lechtenberg, 2007), and laminitis (Cook et al., 2004). Diagnosis of SARA is challenging because of its subacute nature, however, it is typically defined as ruminal pH below 5.6 to 5.8 for multiple h/d (González et al., 2012; Steele et al., 2016; Plaizier et al., 2022). A potentially more accurate measure is the area under the curve (**AUC**) value that calculates not only time spent below the pH threshold but also the severity of the depression (Penner et al., 2009; Schwaiger et al., 2013).

The primary symptom of SARA is low ruminal pH, thus the buffering systems at work within the rumen are of particular interest for mitigating acidosis (Enemark, 2009). Of the neutralization of acids in the rumen, 30% to 40% is thought to be accomplished by buffers in saliva (Allen, 1997). Saliva flow rate peaks during chewing, thus the buffering capacity of saliva is maximized during eating and rumination (Humer et al., 2018), and is influenced by what cattle are fed and how much they consume (Yang and Beauchemin, 2006). The structure and chemical composition of the forage can influence the buffering capacity of the feedstuff (Zebeli et al., 2012). Forage particles stimulate the feeding behaviors of chewing and rumination (Kononoff et al., 2003), which is theorized to increase saliva production to buffer the rumen (Allen, 1997; Maekawa et al., 2002). However, limited literature testing this hypothesis suggests that the influence of increased chewing time on saliva production is minimal (reviewed by Beauchemin, 2018).

Adding to this uncertainty is the mixed evidence that cattle will choose to consume forage when fed high-concentrate diets. Feedlot cattle thwarted from accessing additional beardless wheat hay show equal or less interest in it compared to those prevented from accessing more finisher (82:18, concentrate:forage; Coon and Tucker, 2023a), and there is limited evidence that these animals were more motivated to access alfalfa hay than additional finisher behind an electrified barrier (Coon and Tucker, 2024a). When offered chopped straw separately versus as a total mixed ration (**TMR**), finishing cattle eat less straw than what was fed in the TMR (8% vs. 15%, respectively). However, in contrast, finishing animals allowed free choice of corn silage and barley grain, choose to consume more corn silage than what was fed in the finishing ration (20.4% vs. 10%; Moya et al., 2011, 2014). Adding to the evidence that finishing cattle want more forage, animals fed high-concentrate diets and undergoing an acidosis challenge, will sort in favor of the longer, forage particles in the diet (Dohme et al., 2008; DeVries et al., 2014a). In addition, Van Os et al. (2018) found that feedlot cattle fed high-concentrate diets are motivated to consume Sudan grass hay within minutes of its provision. This mixed evidence about the importance of forage highlights that the factors motivating finishing cattle to consume forage remain unclear, particularly with respect to ruminal pH and the risk of SARA.

Methodological differences among studies also make drawing conclusions about how motivated finishing cattle are to access forage challenging. For example, forages vary in chemical composition and physical structure, as well as particle size within the ration, all of which impact their physical effectiveness (Zebeli et al., 2012) and likely, also their palatability (i.e., the agreeableness of taste and texture). In addition to utilizing a variety of forage types, experiments also differ in whether the risk of acidosis is controlled or not. This could influence the motivation for forage as the severity of SARA is known to be more severe when induced (Plaizier et al., 2022). With this in mind, we chose to measure motivation for Sudan grass hay because the strongest evidence to date exists for this forage type and particle size in feedlot cattle (Van Os et al., 2018).

We have previously measured finishing cattle motivation for alfalfa hay using an electrified barrier (Coon and Tucker, 2024a) and found that this approach distinguishes between the motivation to access a food resource versus nothing. In this work, we also found that finishing cattle were either equally or only slightly more motivated to access alfalfa compared to an additional offering of the TMR they had also been provided ad libitum, for free. This result was surprising, given that we predicted forage would be more valuable for the animals than additional TMR. However, we did not measure any aspect of rumen health, thus were not able to give those findings additional context about why cattle may have made those choices. Introducing the physiological measure of reticulorumen pH may help clarify if internal factors influence how willing cattle are to interact with an electrified barrier to access forage.

The objective of this experiment was to measure how motivated beef steers are to access Sudan grass hay when consuming a high-concentrate finishing diet (referred to here onwards as the “primary” diet) using an increasingly aversive electrified barrier while also measuring reticulorumen pH. When providing Sudan grass hay or an additional offering of the primary diet behind an electrified barrier, we predicted that cattle would be more willing to touch the aversive stimulus with their muzzles at a higher current, approach the treatment more quickly, and visit the treatment more often for the Sudan grass hay, particularly when reticulorumen pH went below pH 5.8 to 5.6.

## Materials and Methods

### Animals and housing

**Figure 1.**
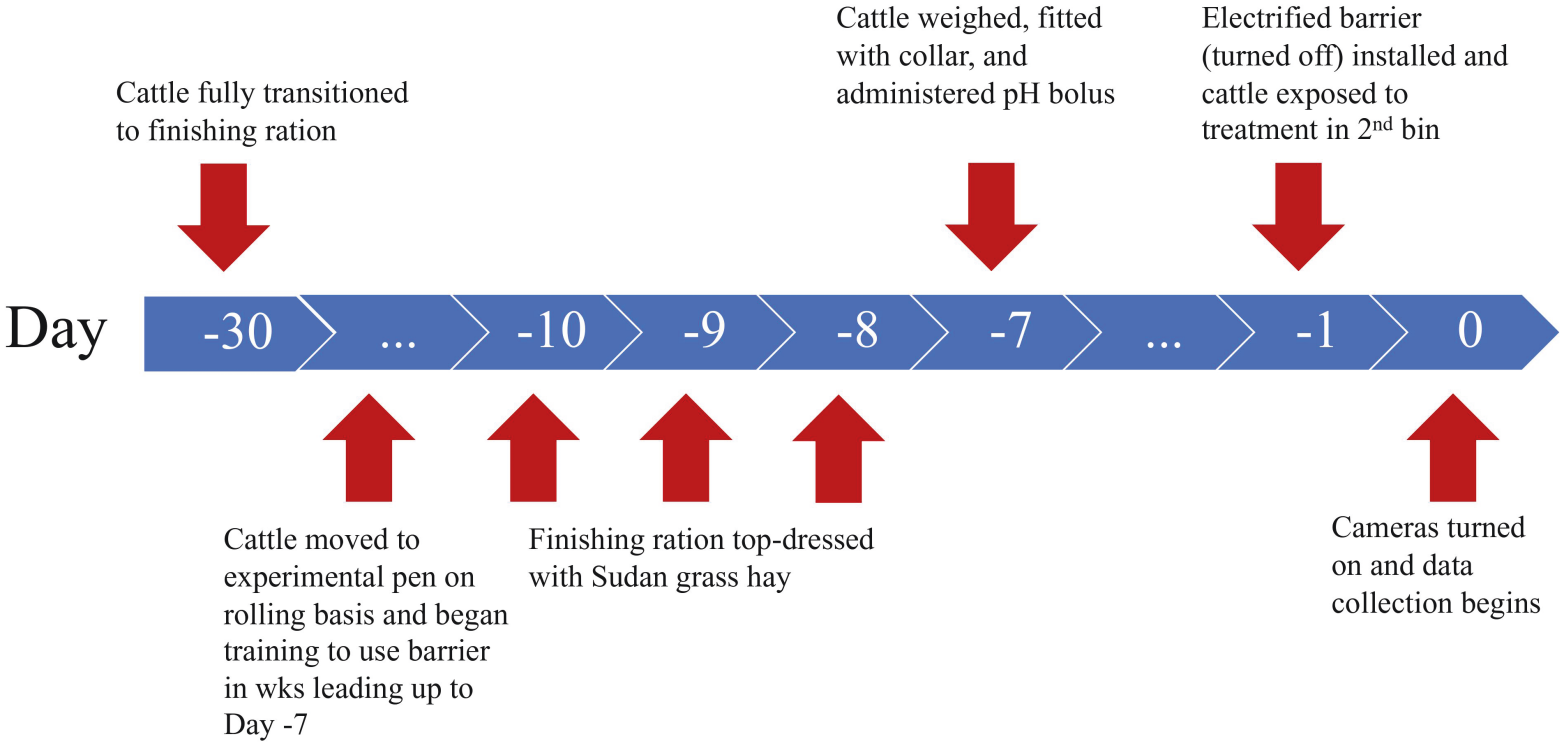
Timeline of days relative to the start of data collection (day 0) for beef steers fed a finishing ration.

All procedures were approved by the University of California Davis Institutional Animal Care and Use Committee (Protocol #21195). The experiment was conducted from November to December 2021. All animals were administered a Revalor-S implant to improve average daily gain (**ADG**) and feed-to-gain efficiency in late August 2021. Twenty-eight Angus-cross steers (11 mo and 475 ± 14 kg; mean ± SD) were housed in groups of eight (30 m^2^/animal) at the University of California Davis feedlot facility in October 2021, before being enrolled in the study. The sample size (*n* = 14 cattle/treatment) was calculated using estimates of variability in maximum price derived from Van Os et al. (2018) to achieve 80% power. The back half of the pens (15 m^2^/animal) were bedded with rice hulls that were scraped and replaced weekly. Animals were fed a finishing TMR (Table 1) into automated feed bins (Insentec B.V., Marknesse, Netherlands) twice daily at 0800 and 1530 h and had ad libitum access to water. After a multi-week step-up protocol from an all-roughage diet to a high-concentrate feed, the finishing ration was fed for a minimum of 30 d (Figure 1) before any behavioral data were collected to allow animals to adapt to a more acidogenic ration (DeVries et al., 2014b). Animals accessed the automated feed bins with an associated RFID ear tag.

**Table 1.** Ingredient and chemical composition (mean ± SD) of SG^1^ and TMR^1^ diet

Composition	TMR	SG
Ingredients, % of DM		
Rolled corn	72.0	—
Sudan hay	—	100
Wheat hay	6.0	—
Alfalfa hay	5.0	—
DDG	6.0	—
Fat	3.0	—
Molasses	3.0	—
Calcium carbonate	1.8	—
Magnesium oxide	0.2	—
Beef trace salt	1.0	—
Urea	1.8	—
Potassium chloride	0.5	—
Rumensin	0.02	—
Chemical composition^2^		
DM, %	83.3 ± 1.7	84.0 ± 4.1
OM, % of DM	94.4 ± 0.6	86.1 ± 1.8
CP, % of DM	14.9 ± 0.5	9.1 ± 1.1
ADF, % of DM	7.7 ± 1.9	44.5 ± 1.4
NDF, % of DM	15.5 ± 1.4	65.8 ± 2.5
NFC, % of DM	63.9 ± 1.7	11.1 ± 4.3
Ca, % of DM	0.7 ± 0.1	0.3 ± 0.02
P, % of DM	0.3 ± 0.02	0.2 ± 0.02
Net maintenance	1.0 ± 0.01	0.4 ± 0.04
Net gain	0.7 ± 0.01	0.2 ± 0.04

^1^SG: Sudan grass hay chopped to 15 cm and TMR: total mixed ration.

^2^Values were obtained from chemical analysis of TMR and SG samples. OM = 100 − % ash. NFC = 100 – (% CP + % NDF + % fat + % ash).

### Training

Animals were moved to an experimental pen in cohorts of four (60 m^2^/animal). Each pen had eight automated feed bins and four rubber mats without any bedding on the ground (15 m^2^/animal) in the lying area. Cattle were trained to access feed behind a barrier hung in front of the opening to the automated feed bins. Animals pushed against the barrier with their muzzles which would rise out of their way, allowing the animal to eat freely from the bin. The training procedures are described in Coon and Tucker (2024a). Cattle were not enrolled in the study until all four animals in the pen were fully trained. All 28 animals successfully learned to use the bins and barriers.

### Experimental design

Approximately 1 wk before enrollment into the study, cattle were administered a wireless telemetry bolus (smaXtec pH Bolus, smaXtec animal care GmbH, Graz, Austria; administration protocol in Figure 1) by R. Coon using a balling gun. The boluses measured reticulorumen pH at 10-min intervals and have been previously validated for measuring reticulorumen pH in cattle (Ammer et al, 2016). At this time, all animals were weighed in a chute with a scale and fitted with a colored collar for identification purposes. Starting on the first day of enrollment and continuing throughout the study, animals were locked in the back half of the pen at 0700 h for 1 h and again at 1500 h for 30 min, preventing them from accessing the feed bins. This allowed for precise measurement of the approach time to the treatments after release. At 0700 h, all bins were carefully cleaned out with a brush and a dustpan before delivering feed. The primary diet was delivered into one of the two side-by-side bins assigned to the animal. The amount of feed offered was adjusted daily, such that it was 115% of the previous day’s intake for each animal to ensure feed was provided ad libitum. Only the primary diet was delivered again during the afternoon feeding.

In the second bin assigned to each animal, one of two treatment diets was distributed during the morning feeding. Fourteen steers were offered 4 L (200 g) of Sudan grass hay (SG) chopped to approximately 15 cm. Fourteen steers were offered 4 L (2,000 g) of the finishing ration (TMR). We chose to match the treatment diets by volume (4 L) instead of weight because 200 g of the TMR was a very small amount of the finishing ration. Preliminary tests showed cattle failed to acknowledge that there was TMR in the feed bin when only 200 g was offered. The heaviest animals were enrolled first in an effort to balance weight gain throughout the finishing period and treatment assignment. Animals were then assigned to the treatments and bins using a random number generator in Excel, ensuring that weight was balanced across treatments. The same was done for assigning treatments to bins within pens without replacement. Treatments and bin assignments were also balanced without replacement across pens. The bin containing the primary diet will be referred to as the “primary bin” and the bin containing the treatment as the “treatment bin”. Animals were released from the back half of the pen at exactly 0800 and 1530 h and allowed to access the feed bins. In an effort to reduce the novelty of the Sudan grass hay, all 28 animals were offered 200 g of Sudan grass hay top-dressed the finishing ration at three consecutive morning feedings before entering the experimental pens (Figure 1).

Before the animals were allowed to interact with the feed bins after moving into the experimental pens, a barrier was hung in front of the treatment bin. The barrier had the capacity to be electrified, but the animal shocker (Precision Animal Shocker, model no. H13-17A, Coulburn Instruments, Holliston, MA, USA) used to electrify the barrier was turned off (Figure 1). Each barrier and associated animal shocker were assessed for functionality twice daily. A detailed description of the barrier, including videos of animals using the device, a list of building materials and dimensions, and instructions for daily functionality assessment can be found in a Dryad repository (Coon and Tucker, 2024b). The barrier was not electrified until the morning feeding of the third day in the experimental pens, at which time a current of 156 µA was applied to the barrier. If the animal continued to successfully access the treatment diet (recorded as a visit to the automated feed bin), the current was increased exponentially the next day to 312 µA. Thereafter, the exponential increase in current every 24 h persisted as long as the animal continued to successfully access the treatment diet. The intervals were 0, 156, 312, 625, 1,250, 2,500, and 5,000 µA. If the animal failed to successfully access the treatment diet at all during the 24-h period, the current was shut off and there was no subsequent increase in current. The animal was considered to have finished the study but remained in the pen until all four animals had reached their maximum currents. The last current at which the animal successfully accessed the treatment diet was deemed the maximum price paid (**MPP**) by that animal.

### Behavioral data collection

The feed bins recorded intake, time spent eating, and visits to both bins. Latency to access the treatment diets following feed delivery and the type of visit (defined below) to the treatment bin were recorded by video cameras (GV-BL4713, GeoVision Inc., Taiwan) positioned directly above each pair of bins. An additional camera recorded the electricity-generating device to monitor functionality throughout the study. GeoVision software (GeoVision Inc., Taiwan) was used to score latency and visit type. A confident visit was defined as an animal that approached the treatment bin, pushed against the barrier, and dislodged it from the magnets. The barrier rose and the animal lowered their head into the bin for more than 1 s. The definition of an unsuccessful attempt was that an animal approached the treatment bin, pushed against the barrier but failed to dislodge it from the magnets. The barrier did not rise, and the animal was unable to access the bin. Video examples of each behavior type are available online (https://doi.org/10.25338/B8HW7R, Coon and Tucker, 2024b). We anticipated that these metrics would change as the electric current increased, with fewer confident visits and more unsuccessful attempts demonstrating greater hesitancy to interact with an aversive stimulus.

Eight observers were reliably trained to score the videos. A minimum kappa (kappa2 function in irr package, version 0.84.1) statistic for interrater reliability of 0.80 was required when compared with R. Coon to proceed with video coding. To calculate their kappa score for each behavior, each observer coded 24 videos that were up to 1 min 45 s in length and included at least one confident visit or unsuccessful attempt to access the treatment bin. The observer coded the video if the type of visit (confident or unsuccessful) matched the type R. Coon had identified for the same visit. To meet the assumptions of Cohen’s kappa statistical test, each visit was nominal (categorized), the outcome variable (visit type) had the exact same categories between the observer and R. Coon, and the videos (observations) were paired between the observer and R. Coon.

### Feed sampling and analysis

Once per week, a fresh feed sample of the TMR and the SG was collected and frozen immediately at −20 °C until further analysis. Samples were sent to Cumberland Valley Analytical Services Inc. (Maugansville, MD) for analysis of DM (135 °C; AOAC International, 2000: method 930.15), ash (535 °C; AOAC International, 2000: method 942.05), ADF (AOAC International, 2000: method 973.18), NDF with heat-stable α-amylase and sodium sulfite (Van Soest et al., 1991), and CP (*N* × 6.25; AOAC International, 2000: method 990.03; Leco FP-528 Nitrogen Analyzer, Leco, St. Joseph, MI).

### Statistical analyses

A total of 6 animals (five SG and one TMR) were removed from analyses because they did not visit their treatments during the 24 h before the start of the experiment. Another TMR animal was removed from analyses because of human error. Nine 24-h periods were removed from various animals’ data (including behavioral, intake, and pH) and subsequent analyses due to technological failure. Methods for avoiding these failures in future research are provided in supplementary Materials for Coon and Tucker (2024b). Two animals (one SG and one TMR) have no intake data from their primary bins because of technological failure. All analyses were conducted using Rstudio (ver. 4.2.0) on macOS 12.4.

The proportion of steers successfully accessing treatments at each electrical current level was analyzed using a survival analysis (logrank) conducted with the Surv, survfit, and ggsurvplot functions in the survival (version 3.3-1) and survminer (version 0.4.9) packages. A Wilcoxon rank sum test was used to test for differences between treatments for MPP using the wilcox.test function in the stats package (version 3.6.2).

To control for differences in MPP, the remaining measurables were computed in terms of days leading up to the maximum current touched with period −1 being the 24-h period before the day in which the animal reached their MPP (period 0). All repeated measures models analyzed treatment differences from current periods −3 to 0 and used measurements from each period for each animal as the repeated measure.

The amount of either SG or TMR from the treatment bin consumed during a 24-h period was summed for each animal and divided by the total amount offered. Treatment differences in intake (% of offered amount) of either SG or TMR for periods −3 to 0 were analyzed using a generalized linear mixed model, with treatment and period and their interactions as fixed effects and animals as random effects. Due to data being proportions, a beta distribution was chosen (family = beta_family(link= “logit”)) with the glmmTMB function. The DHARMa package (version 0.4.5) in R was used to plot residuals and test for over-dispersion by utilizing the testDispersion function.

Differences in the number of confident visits and unsuccessful attempts as well as visits registered by the Insentec RIC system to the treatment bin were compared between treatments for periods −3 to 0 using a generalized linear mixed model with treatment and period and their interactions as fixed effects and animal as random effects. The number of visits to each category was summed by period for each animal. The models were fit using the glmmTMB function from the glmmTMB package (version 1.1.4), treating a number of visits as count data and fitting a Poisson model (link=“log”). The DHARMa package was again used to plot residuals and tested for over-dispersion. Estimated marginal means were calculated for contrasts for the intake and visit models using the emmeans function (emmeans package, version 1.4.5). These values were back-transformed from the logit and log scales for plotting purposes using the “type=‘response’” option.

Latency was calculated as the time in minutes to access the treatments after animals were released at 0800 h each day. These latency data did not meet assumptions of a linear model despite transformations and were analyzed using four Wilcoxon Rank Sum tests for periods −3 to 0 using the function wilcox.test in the stats package (version 3.6.2).

Absolute values for areas below a pH threshold of 5.8 and 5.6 (AUC < pH 5.8, 5.6) were calculated using methods described in Coon et al. (2018). Repeated measures correlation analyses were conducted between the daily AUC values and the number of confident visits, unsuccessful attempts, RIC registered visits, latency to approach, and percent intake from the treatment bin for each treatment separately. The rmcorr function in the rmcorr package (version 0.5.4) was used to conduct the analyses with a confidence level of 0.95, using animal ID as a random effect.

The following measures were calculated for descriptive purposes only. The intake from the primary bin, the number of RIC registered visits to the primary bin, and the total time spent eating from both the primary and treatment bins were summed daily for each animal. Treatment means and standard errors (**SE**) were then calculated. The same metrics were calculated for the daily intake in kg of SG and TMR from the treatment bins. The mean, maximum, and minimum reticulorumen pH were averaged (±SE) by treatment, as was the daily time spent below pH 5.8 and 5.6. Finally, the treatment averages (±SE) for AUC < pH 5.8, AUC < pH 5.6, AUC standardized by DMI (AUC < pH 5.8/DMI; (Penner et al., 2009), and AUC < pH 5.6/DMI were calculated from each animal’s daily averages.

## Results

The data (https://doi.org/10.25338/B88D37, Coon and Tucker, 2024c) and RMarkdown files including the means, SE, and confidence intervals for all raw data and back-transformed model predicted means (https://doi.org/10.5281/zenodo.8030121, Coon and Tucker, 2024d), well as supplementary materials, including Tables S1 and S2, which contain test statistics and model degrees of freedom (https://doi.org/10.5281/zenodo.8030125, Coon and Tucker, 2024e) can all be found online. Interactions of treatment and period were only described when *P* ≤ 0.10. The sample sizes for all inferential analyses were 9 SG and 12 TMR animals.

### Descriptive results

**Figure 2.**
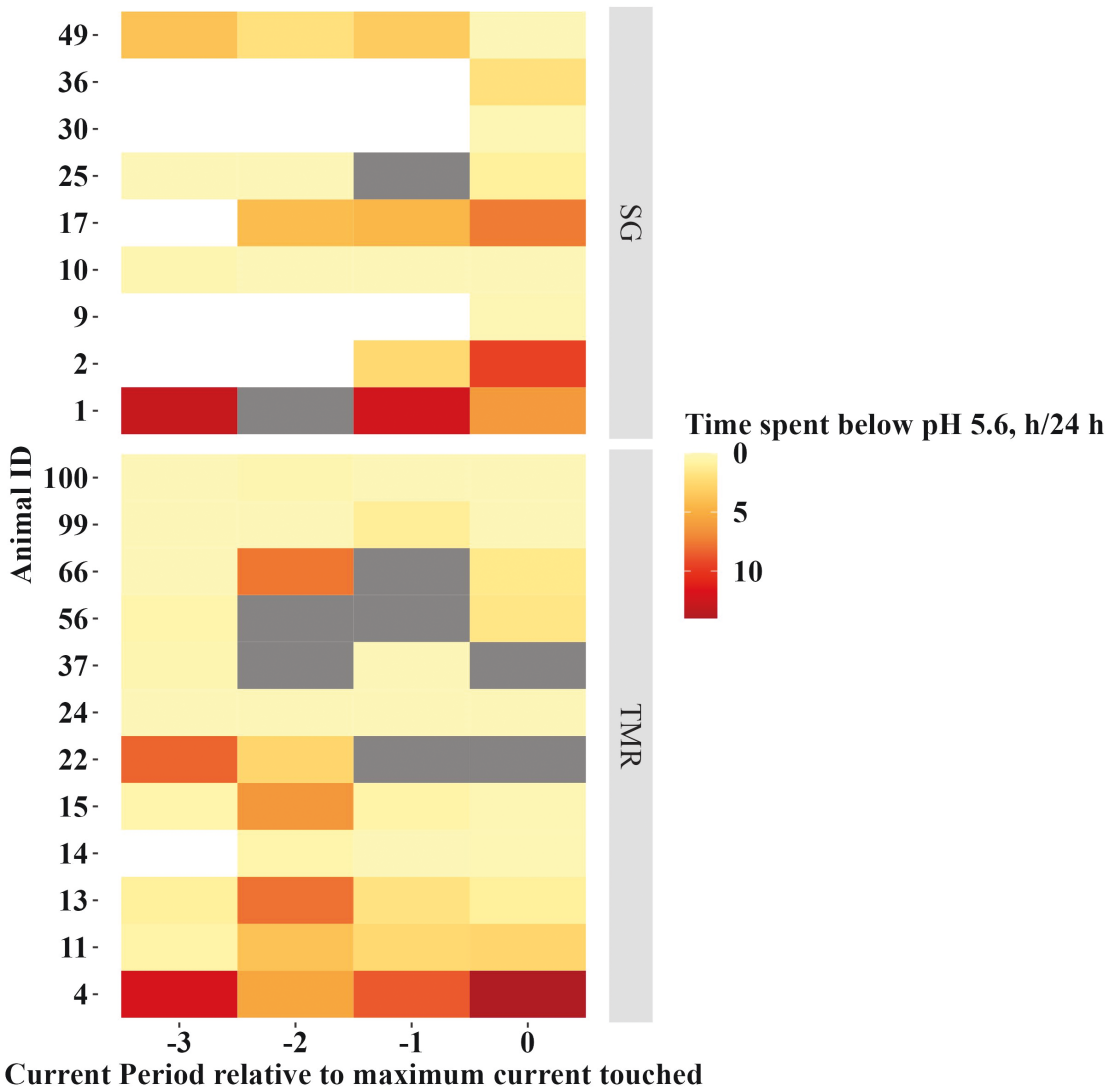
The time spent below reticulorumen pH 5.6 (h/24 h) for finishing steers plotted relative to the 24-h period during which they successfully accessed a dietary treatment by touching an electrified barrier. In additional to being fed the high-concentrate diet, cattle had access to one of two treatments: 1) Sudan grass hay chopped to approximately 15 cm (SG; *n* = 9) or 2) an additional offering of the finisher ration (TMR; *n* = 12). Periods were 24-h intervals during which the current (μA) applied to the electrified barrier remained constant. If the animal continued to successfully access their respective treatment, the current was increased exponentially every 24 h. Period 0 is the 24-h interval when the animal reached their highest maximum current while period −1 is the 24-h interval before that and so on. Gray boxes indicated data removed from analyses and white boxes indicate that an animal did not progress to that stage when the current was increased. This figure is in color in the online version.

The mean daily intake, time spent eating from, and visits to the primary bin are reported in Table 2. The mean (±SE) daily intakes from the treatment bin were 84.5 ± 18.1 and 1,294.0 ± 52.9 g for SG and TMR animals, respectively. The daily mean reticulorumen pH, as well as the mean maximum and minimum pH values for both treatments, are reported in Table 3. The mean daily time spent below pH 5.8 and 5.6, mean daily AUC < pH 5.8, AUC < pH 5.6, and mean daily AUC < pH 5.8/DMI and AUC < pH 5.6/DMI can also be found in Table 3. The daily time spent below reticulorumen pH 5.6 for each animal has been plotted by period and animal ID in Figure 2.

**Table 2.** Descriptive effects of interacting with an electrified barrier to access either 4 L of SG^1^ (200 g) or TMR^1^ (approximately 2 kg) fed once daily on intake and behavioral responses (mean ± SE) in beef cattle

Behavior	Treatment	
	SG	TMR
Primary bin		
Dry Matter Intake (DMI), kg/24 h	9.4 ± 0.8	9.1 ± 0.2
RIC registered visits^2^^,^ #/24 h	17.3 ± 2.6	20.4 ± 2.4
Both bins (primary and treatment)		
Total DMI, kg/24 h	9.8 ± 0.8	10.4 ± 0.3
Total time spent eating, min/24 h	62.4 ± 6.6	73.0 ± 4.1

^1^SG: Sudan grass hay chopped to 15 cm and TMR: total mixed ration.

^2^Visits to the primary bin as recorded by the automated feed bin.

**Table 3.** Descriptive effects of interacting with an electrified barrier to access either 4 L of SG^1^ (200 g) or TMR^1^ (approximately 2 kg) fed once daily on reticulorumen pH (mean ± SE) in beef cattle

Item	Treatment	
	SG	TMR
Mean reticulorumen pH/24 h	6.1 ± 0.1	6.1 ± 0.1
Maximum reticulorumen pH/24 h	6.7 ± 0.1	6.6 ± 0.0
Minimum reticulorumen pH/24 h	5.4 ± 0.1	5.5 ± 0.1
Mean time below reticulorumen pH 5.8, min/24 h	348 ± 101	280 ± 76
AUC^2^ pH < 5.8, pH × min/24 h	87.0 ± 33.4	66.2 ± 24.2
AUC^2^ pH < 5.8/DMI^3^	7.1 ± 2.7	6.7 ± 2.8
Mean time below reticulorumen pH 5.6, min/24 h	179.2 ± 71.9	138.5 ± 51.1
AUC^2^ pH < 5.6, pH × min/24 h	34.1 ± 17.3	24.3 ± 12.2
AUC pH < 5.6/DMI^4^	2.0 ± 1.0	2.5 ± 1.4

^1^SG: Sudan grass hay chopped to 15 cm and TMR: total mixed ration.

^2^Area Under Curve.

^3^AUC/DMI = AUC pH < 5.8 (pH × min/24 h) divided by DMI (kg/24 h).

^4^AUC/DMI = AUC pH < 5.6 (pH × min/24 h) divided by DMI (kg/24 h).

### MPP and survival analyses

**Figure 3.**
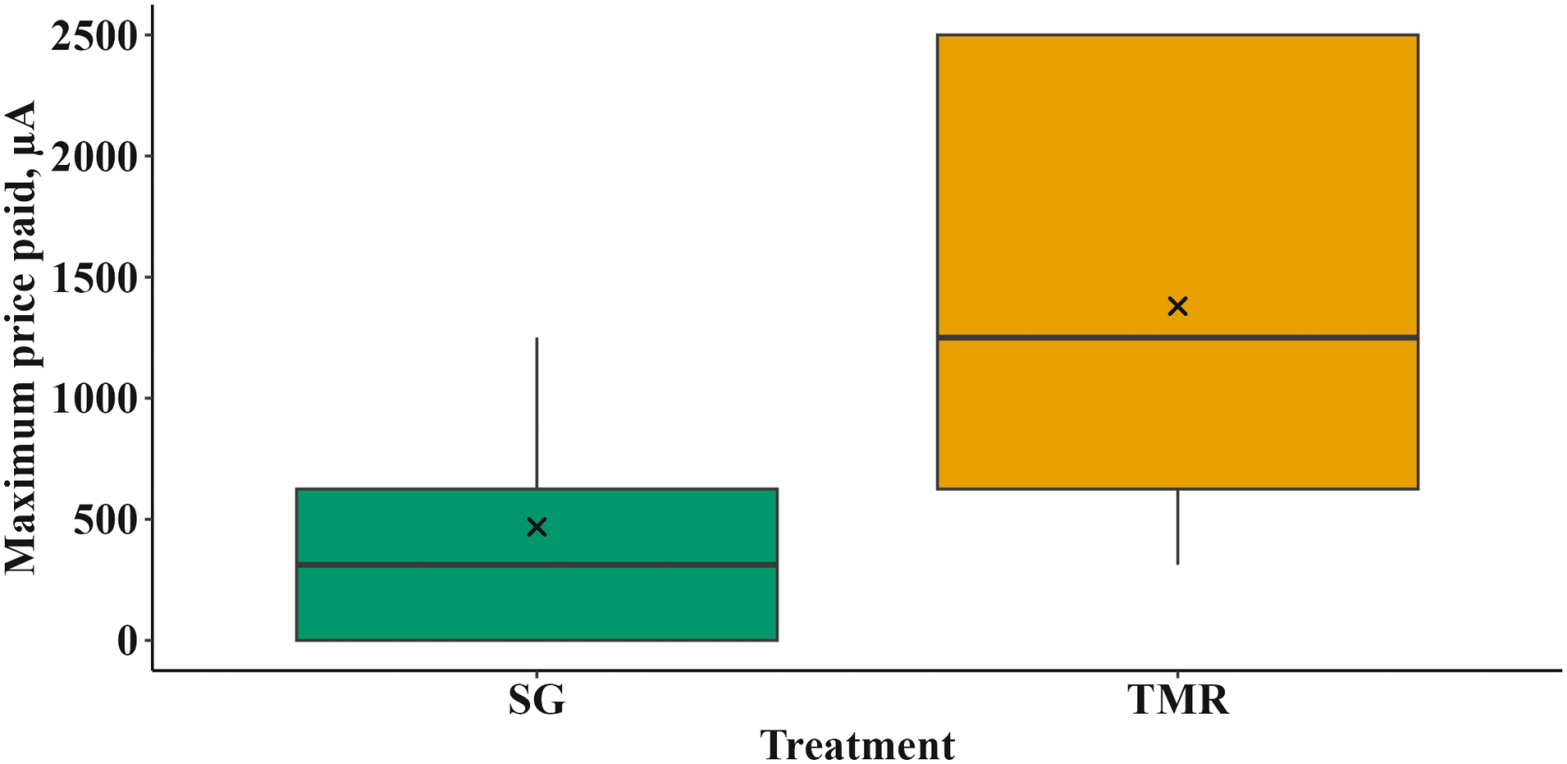
The maximum current (μA) at which finishing steers successfully accessed their treatments fed behind an electrified barrier. The treatments were composed of SG: Sudan grass hay chopped to 15 cm (*n* = 9), and TMR: total mixed ration (*n* = 12). The median is represented by a black line within the box and the boxes contain the first and third quartiles (25% and 75% of data). Outliers (1.5× the interquartile range) are identified as black dots and the means as “×”.

**Figure 4.**
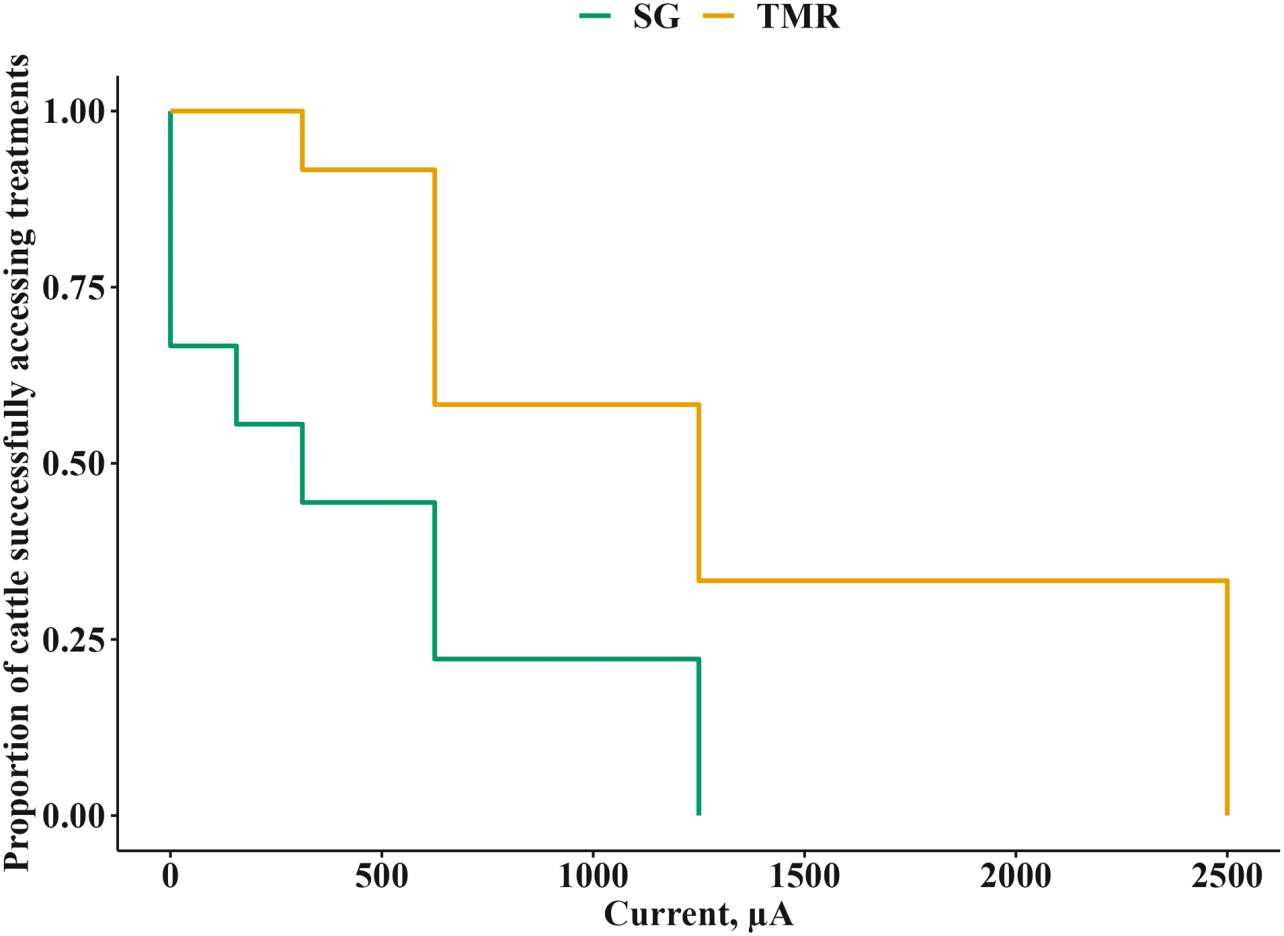
Kaplan–Meier curve showing the proportion of finishing cattle who continued to successfully access the treatments behind an electrified barrier as the current (μA) increased exponentially. The treatments were composed of SG: Sudan grass hay chopped to 15 cm (*n* = 9), and TMR: total mixed ration (*n* = 12). This figure is in color in the online version.

The MPP by TMR animals was almost three times as high as that paid by SG animals (*P* = 0.01; Figure 3). The probability of advancing to the next current level was also higher for TMR animals than SG animals (*P* < 0.01; Figure 4). While sample sizes were reduced (TMR, *n* = 12; SG, *n* = 9), a post hoc sample size calculation using the common standard deviation of the two treatment means revealed that to detect a 30% difference in MPP, each treatment needed five individuals. This indicates that the analysis had sufficient power for the primary outcome despite the reduced sample sizes.

### Percent intake

**Figure 5.**
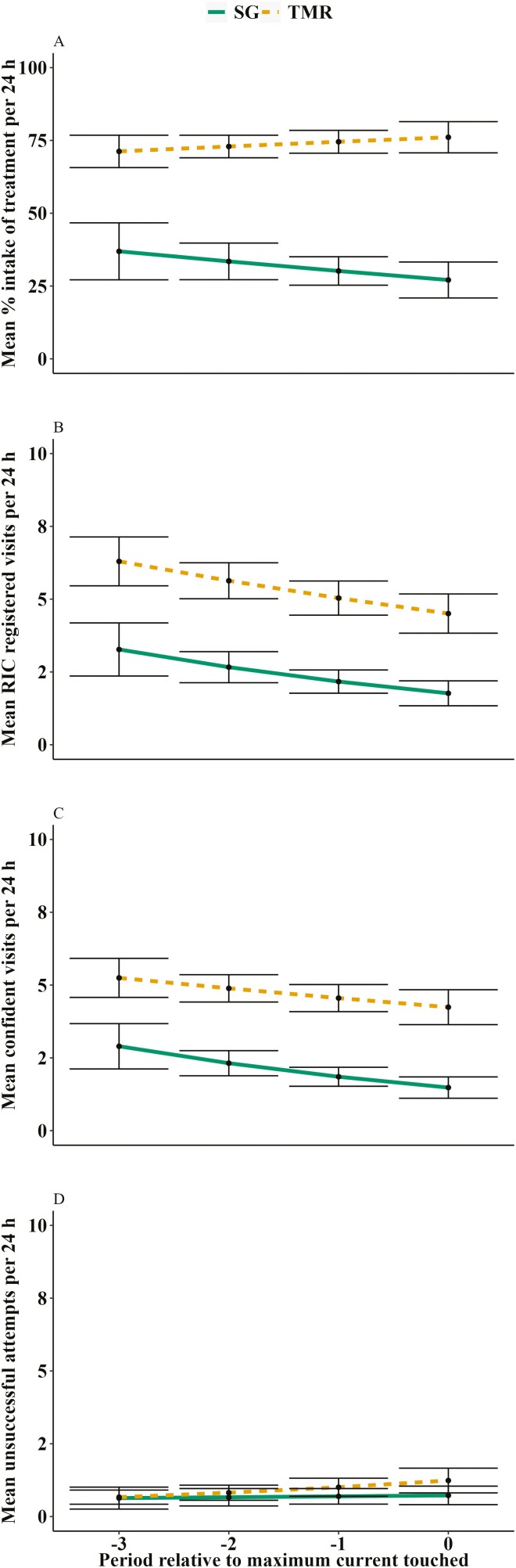
Behavioral differences between feedlot cattle offered one of two treatments behind an electrified barrier to determine motivation for forage: 1) SG: Sudan grass hay chopped to 15 cm (*n* = 9) and 2) TMR: total mixed ration (*n* = 12). Periods were 24-h intervals during which the current (μA) applied to the electrified barrier remained constant. If the animal continued to successfully access their respective treatment, the current was increased exponentially every 24 h. Period 0 is the 24-h interval when the animal reached their highest maximum current while period −1 is the 24-h interval before that and so on. A) Mean intake of the treatments as a percentage of the amount offered behind the barrier, B) mean visits to the bin that were registered by the Insentec RIC system, C) mean number of confident visits to the treatment bin, D) mean number of unsuccessful attempts to the treatment bin. A confident visit was defined as an animal that approached the treatment bin, pushed against the barrier and dislodged it from the magnets. The barrier rose and the animal lowered their head into the bin for more than 1 s. The definition of an unsuccessful attempt was that an animal approached the treatment bin, pushed against the barrier but failed to dislodge it from the magnets. Model predicted means (±SE) presented for all figures.

Animals fed TMR consumed more of their treatment as a percentage of what was offered than SG animals (*P* < 0.01; Figure 5A), regardless of time.

### Visits

According to the Insentec RIC system, TMR animals visited their treatment more than twice as often as SG animals (*P* < 0.01; Figure 5B). The number of confident visits was also greater for TMR animals (*P* < 0.01; Figure 5C), although confident visits tended to decline for both treatments as the current increased (*P* = 0.09). There was no evidence that the number of unsuccessful attempts to access food resources differed between treatments (*P* = 0.32; Figure 5D).

### Latency

**Figure 6.**
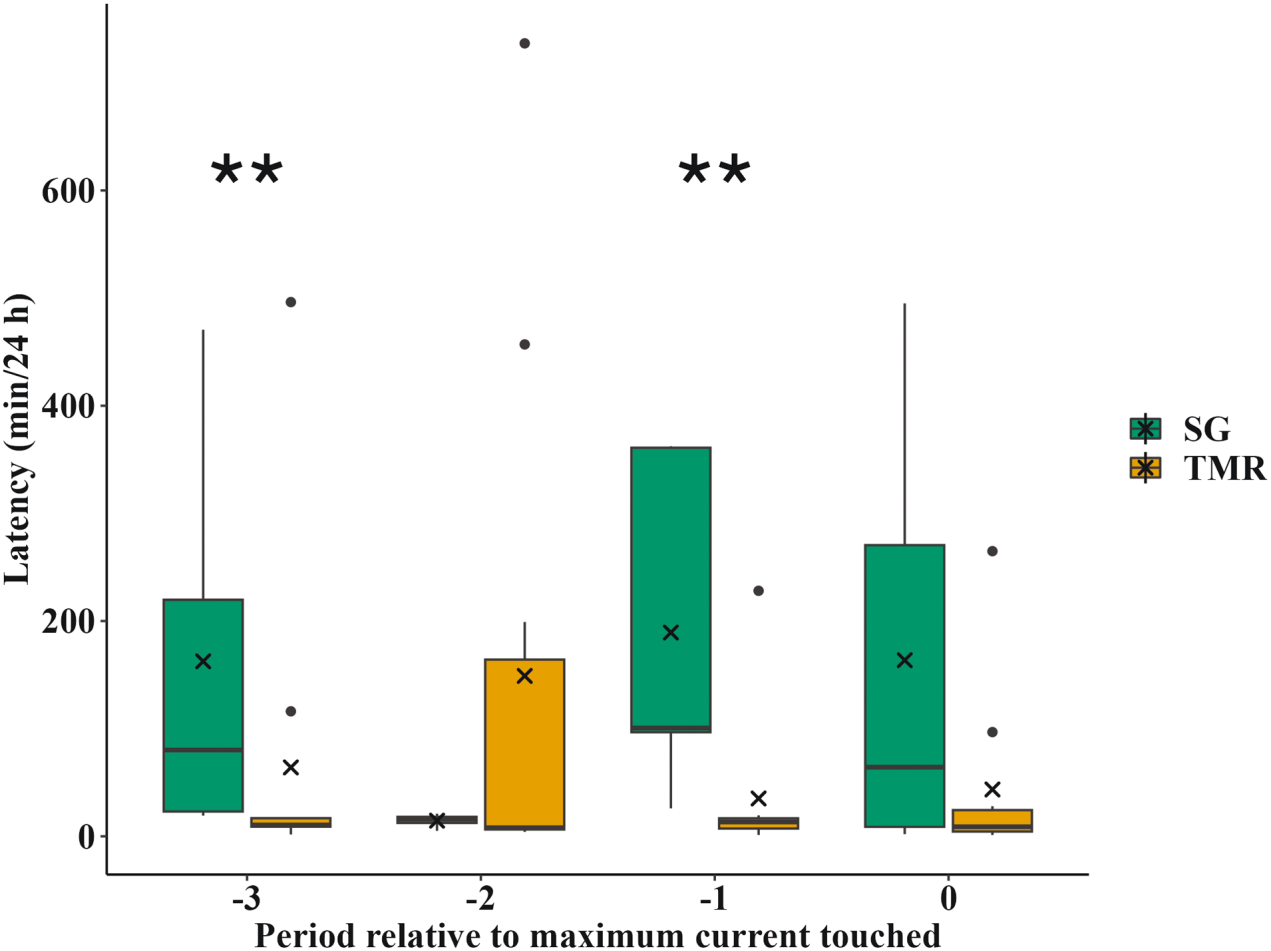
The mean latency in minutes for finishing cattle to access treatments behind an electrified barrier. The treatments were composed of 1) SG: Sudan grass hay chopped to 15 cm (*n* = 9) and 2) TMR: total mixed ration (*n* = 12). Periods were 24-h intervals during which the current (μA) applied to the electrified barrier remained constant. If the animal continued to successfully access their respective treatment, the current was increased exponentially every 24 h. Period 0 is the 24-h interval when the animal reached their highest maximum current while period −1 is the 24-h interval before that and so on. The median is represented by a black line within the box and the boxes contain the first and third quartiles (25% and 75% of data). Outliers (1.5x the interquartile range) are identified as black dots and the means as “x”. Asterisks indicate significant difference between treatments (** for *P* ≤ 0.05).

Animals offered TMR were quicker to approach their treatments during periods −3 and −1 (*P* < 0.04; Figure 6), but there was no evidence that the treatments differed in their latency to approach during periods −2 and 0 (*P* ≥ 0.13).

### Area under the curve for pH

**Figure 7.**
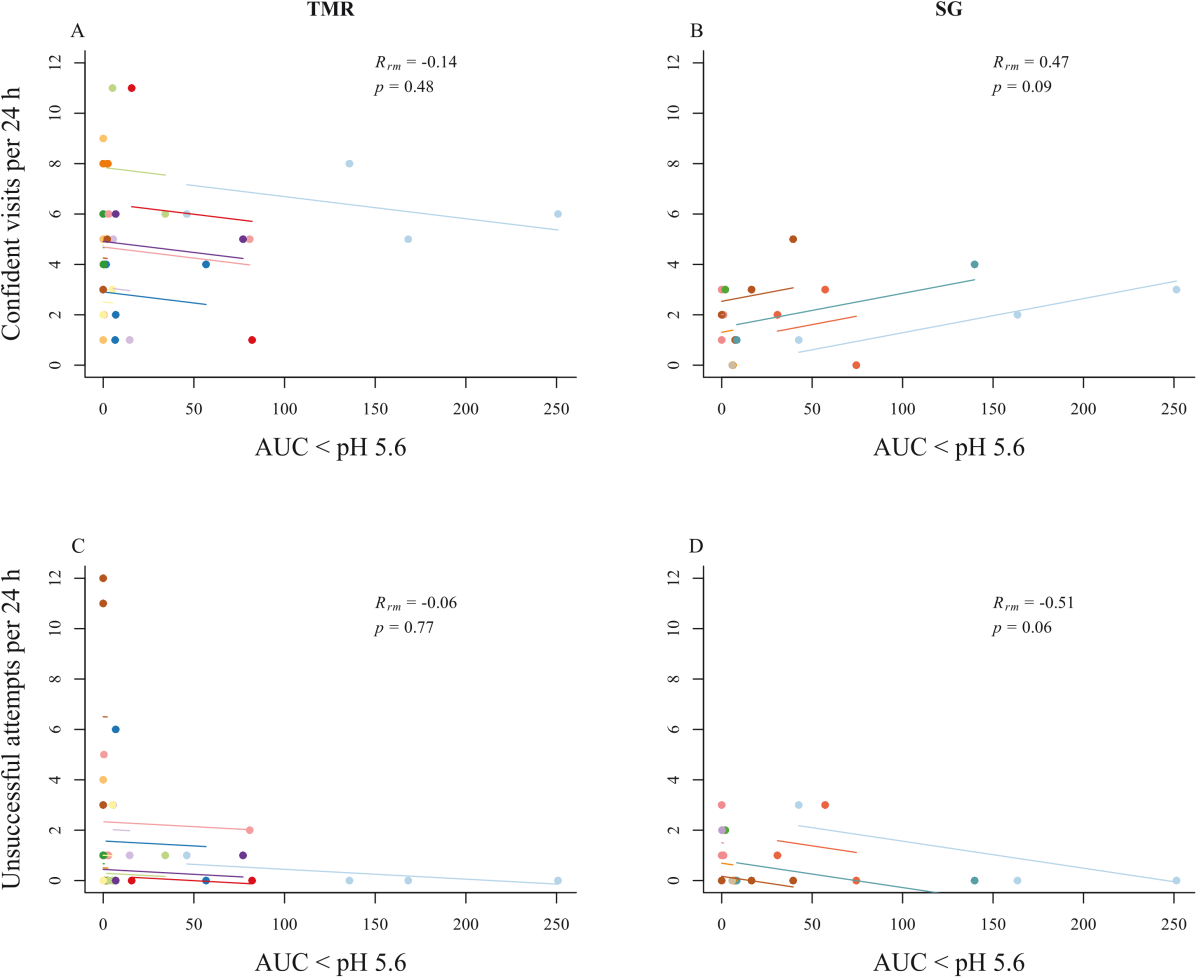
Correlation (*r*) of AUC below reticulorumen pH 5.6 and two types of visits (confident visits and unsuccessful attempts) to treatments offered behind an electrified barrier by finishing cattle fed high-concentrate diets. In addition to being fed the high-concentrate diet, cattle had access to one of two treatments: 1) Sudan grass hay chopped to approximately 15 cm (SG; *n* = 9) or 2) an additional offering of the finisher ration (TMR; *n* = 12). Correlation plot of AUC and the number of confident visits per 24 h in TMR cattle (A) and in SG cattle (B), and the number of unsuccessful attempts per 24 h in TMR cattle (C) and in SG cattle (D). Colors represent individual animals and figure is in color in the online version.

There was no relationship detected between the number of confident visits and AUC for TMR cattle (AUC < pH 5.8: *r* = −0.2, *P* = 0.31, AUC < pH 5.6: *r* = −0.1, *P* = 0.47; Figure 7A), although there was a tendency for a positive correlation for SG cattle (AUC < pH 5.8 and 5.6: *r* = 0.5, *P* = 0.09; Figure 7B). There was no correlation between AUC and unsuccessful visits for TMR cattle (−0.06 ≤ *r* ≤ −0.01, *P* ≥ 0.77; Figure 7C). The number of unsuccessful attempts was negatively correlated with AUC < pH 5.8 for SG cattle (*r* = −0.6, *P* = 0.04) and there was also a tendency for this to occur when AUC < pH 5.6 (*r* = −0.5, *P* = 0.06; Figure 7D). The remaining feeding behaviors (RIC registered visits, latency, and percent intake of the treatments) were not correlated to either daily AUC pH < 5.8 or daily AUC pH < 5.6 (−0.14 ≤ *r* ≤ 0.4; *P* ≥ 0.13).

## Discussion

Despite widespread evidence of prolonged daily reticulorumen pH depression in this experiment, overall, steers were less motivated to access additional forage compared to additional TMR. Not only was the MPP by SG animals lower, but they were also slower to approach their treatment following feed delivery, visited less often, and consumed less of it as a percentage of what was offered compared with TMR animals. The reticulorumen pH was on average, below 5.6 for approximately 2.3 h/ 24 h for TMR animals and 3 h/24 h for SG animals, which meet the criteria for diagnosis of SARA (González et al., 2012). Indeed, the severity of the pH depression tended to be correlated to more confident visits and fewer unsuccessful attempts to access SG, but not TMR, suggesting we have some evidence that cattle may have been trying to access more forage to offset SARA.

Overall, animals visited the SG treatment less often, consumed less of it and had a longer latency to access the forage in periods −3 and −1. There were also five SG steers that dropped out before data collection of these measures began, compared to only one TMR animal. One potential explanation for lower motivation for SG could be that animals were given a minimum of 30 d to acclimate to the finishing ration. Finishing cattle will adjust their sorting behavior to increase their intake of physically effective fiber after an acidosis challenge when fed a high-concentrate diet for 34 d leading up to it, while animals with only 8 d to acclimate do so to a lesser degree (DeVries et al., 2014b). If our animals were also engaging in this behavior, they may have been consuming approximately 1.2 kg of forage regardless of treatment, however, sorting behavior was not measured, nor was it observed anecdotally.

The primary diet was denser in calories and likely highly palatable, which could also have contributed to a higher MPP and greater willingness to touch higher currents in TMR compared to SG cattle. These results could be explained by Optimal Foraging Theory (**OFT**). The theory predicts that animals will consume the most energetic diet (Sih and Christensen, 2001). By matching by volume instead of weight, there was approximately 10 times as much TMR offered as SG behind the barrier, which despite all animals being fed ad libitum, might have influenced the attractiveness of the TMR over the SG. When factoring in OFT and the disproportionately larger offering of TMR, it is logical that animals who have been bred for greater ADG and thus higher DMI would be more motivated to access the TMR (Haskell et al., 2019). This may be particularly salient for this population, where before the start of the experiment, all animals were administered Revalor-S, an implant designed to increase feed efficiency and known to stimulate greater intake (Hermesmeyer et al., 2000). These factors, in combination with the highly palatable and calorically dense TMR, could explain the contrafreeloading TMR cattle exhibited in both this experiment and a previous study (Coon and Tucker, 2024a). There are more easily fermentable carbohydrates in the TMR than the SG and sugar has been shown to promote addictive-like behaviors in rodents (Avena et al., 2005; Wei et al., 2021), although it has not been studied in this context in cattle. While it might have been expected that finishing cattle would seek out forage given the consequences of SARA, animals do not always make choices in their best interests (Fraser and Nicol, 2011), and energetic value and palatability appear to have taken precedence in this population to some degree. Contrafreeloading of this magnitude for a high-concentrate ration is unprecedented and is particularly striking because it was observed despite an aversive stimulus and high average AUC values. Considering that cattle exhibit avoidance behavior when 6,000 µA is applied to the rump (Whittlestone et al., 1975) and aversion to electrical current is well documented in cattle (e.g., Pajor et al., 2000; reviewed by Grumett and Butterworth, 2022), it is compelling that TMR animals willingly touched up to 2,500 µA to contrafreeload.

Part of the animals’ attraction to the TMR may be explained by its familiarity relative to the SG, as cattle were fed the finishing ration for at least 30 d before the experiment began. Animals may have been averse to the SG because they had only been introduced to it a few times and cattle are known to be neophobic of novel foods (Mainardes and DeVries, 2016; Van Os et al., 2017; Morrow et al., 2023). Neophobia towards the SG could also explain why five SG steers and only one TMR steer were removed from the analyses for failing to access forage during the 24 h before the start of the experiment. Offering more time to acclimate to the SG might have increased motivation for roughage since neophobia can be overcome with repeated exposure in this species (Herskin et al., 2003; Mainardes and DeVries, 2016). Additional exposures to the SG would potentially have also allowed cattle more time to discover its ameliorating benefits for SARA if 200 g daily is sufficient to do so. However, the time necessary for cattle to learn this characteristic of forage has not been studied and likely depends on the animal, the severity of SARA, and the forage’s physical and chemical structure. While Van Os et al. (2018) found that cattle were highly motivated to obtain SG, half of those animals had been fed a diet composed entirely of SG for at least 30 d prior. For those animals who did not have 30 d to acclimate, the SG was used during both the training and testing portions of the experiment (Van Os et al., 2018). This may have resulted in more exposure to SG than in the present experiment where this hay was only offered for 3 d before entering the experimental pen top-dressed on the primary diet. The only exposure cattle had to SG *behind a barrier* before the experiment began was the 24-h period before behavioral observations commenced, meaning both the feed and its presentation were relatively novel at the time of testing. Herskin et al. (2003) observed that a combination of multiple exposures and increased palatability accelerated cattle’ acclimation to novel feeds, which supports the evidence that animals were more motivated for a familiar grain-based diet with a high starch content than for a novel forage.

All animals likely consumed some forage, either in the primary diet or as additional SG, and this intake may have been influenced by the chronic and severe low reticulorumen pH experienced by cattle in both treatments. The primary diet contained 11% roughage on a DM basis, which when combined with the 200 g of SG offered daily behind the electrified barrier, means SG animals received approximately 1.2 kg/24 h from roughage sources alone, assuming they consumed 9.4 kg (mean daily DMI for SG animals) of their primary diet and 200 g of the treatment. When finishing bulls were offered straw and pelleted concentrate separately, they consumed less straw (8:92, straw:concentrate, or approximately 0.9 kg/24 h) than was predicted for bulls fed a TMR (15:85, or 1.7 kg/24 h) without increasing their risk of rumen ulcers (Genís et al., 2021). Increased roughage intake by animals fed the TMR led to higher mean ruminal pH in those animals (Genís et al., 2021). There is also evidence that cattle will ingest *more* corn silage when the components of the diet are offered instead of as a TMR; finishing cattle consumed approximately 1.7 and 1.3 kg/24 h of silage (Moya et al., 2011, 2014), versus 0.9 kg offered in the TMR. When the latter results are combined with the previous research demonstrating high motivation for Sudan grass hay in finishing cattle (Van Os et al., 2018) and the weak evidence for higher motivation for alfalfa hay than TMR (Coon and Tucker, 2024a), it seems unlikely that the 11% forage inclusion was adequate, especially in light of widespread low reticulorumen pH.

There is individual variation in how susceptible cattle are to acidosis when fed a high-concentrate diet, and their behavior towards forage is different depending on their risk (Gao and Oba, 2014; Coon et al., 2019). Animals in the current study differed in their susceptibility to SARA, but perhaps because the risk was likely balanced across treatments and forage consumed was approximately equal for SG and TMR animals, motivation for SG was not higher overall. However, these results are different when AUC for pH 5.8 and 5.6 are incorporated into the analyses of motivation.

Animals with access to SG showed more confident visits and fewer unsuccessful attempts to access forage as the severity of low reticulorumen pH increased. These correlations support the findings that finishing cattle seek out longer forage particles when experiencing acidosis (DeVries et al., 2014b). In comparison, animals with access to TMR did not change their behavior towards additional offering of it, despite experiencing equally severe reticulorumen pH depression. While pH was not measured in the previous experiments measuring motivation for forage in feedlot cattle, these findings agree with the strong evidence for high motivation for SG found by Van Os et al. (2018) and the weak evidence found for higher motivation to access alfalfa hay than TMR in Coon and Tucker (2024a).

## Conclusions

Overall, animals worked harder to access TMR than SG, interacting with higher currents, visiting more often, and consuming more of the treatment as a percentage. Differences in quantity, palatability, and familiarity of the two treatments may have made the TMR more appealing than the SG. Given that contrafreeloading has now been observed for both high and low-roughage diets, future research should investigate if there are alternative explanations beyond dietary selection for finishing cattle’ propensity to work for what is already freely available to them. While these measures demonstrate that cattle were motivated to access TMR, the findings of the reticulorumen pH monitoring indicate the severity of pH depression is related to motivation for forage, more so than TMR. Cattle tended to access SG more often and showed fewer unsuccessful attempts to interact with an aversive electrified barrier as pH depression became more severe. These correlational results are supportive of previous work showing finishing cattle seek out additional forage when experiencing SARA.

Acknowledgements

We thank the University of California Davis Feedlot Facility manager, Marissa Fisher, feedlot residents, and the undergraduate interns for animal care and support. Thanks to Dr. Karen Schwartzkopf-Genswein and Dr. Kristina Horback for their expertise and feedback on earlier version of this manuscripts. We are grateful to Wen-Chi (Nina) Chiu, who assisted with all aspects of data collection. We are also thankful for the infrastructure support of the Department of Animal Science, College of Agricultural and Environmental Sciences, and the UC Davis California Agricultural Experiment Station. This study was supported by the USDA Multistate Research Project NC1029 and the University of California Davis Smart Farm Initiative.

## Abbreviations

AUC: area-under-the-curve
BW: body weight
DM: dry matter
MPP: maximum price paid
RFID: radio frequency identification
SARA: subacute ruminal acidosis
SG: Sudan grass hay
TMR: total mixed ration
